# Epithelial–Mesenchymal Transition in Human Alveolar Cells Exposed to Indium Chloride

**DOI:** 10.1002/jat.4848

**Published:** 2025-07-07

**Authors:** Eiki Kimura, Sharif Ahmed, Haijiao Chen, Yusuke Hiraku

**Affiliations:** ^1^ Department of Environmental Health University of Fukui School of Medical Sciences Eiheiji Japan

**Keywords:** epithelial–mesenchymal transition, human alveolar cells, indium chloride, indium lung disease, occupational exposure

## Abstract

For workers in the industry, occupational exposure to indium compounds induces pulmonary disorders, such as interstitial pneumonia. Moreover, lung cancer has been reported in both humans and rodents exposed to indium compounds by inhalation. However, the biological mechanism underlying indium‐induced disorders is poorly understood. Epithelial–mesenchymal transition (EMT)—the cellular process of losing epithelial and acquiring mesenchymal characteristics—is linked to fibrosis and cancer progression. Therefore, we examined whether indium exposure elicits EMT in vitro. A549 human alveolar epithelial cells treated with indium chloride at doses of 0–500 μg/mL for 24 h were used to analyze EMT marker expression and cytoarchitecture. Significant downregulation of *CDH1* mRNA expression as an epithelial marker after treatments at 125, 250, and 500 μg/mL occurred dose‐dependently; conversely, the mesenchymal marker *SNAI1* was upregulated. Consistent with mRNAs, the expression levels of EMT marker proteins (i.e., E‐cadherin, ZO1, SNAIL, and Vimentin) were changed significantly by treatment. While NF‐*κ*B signaling was activated in treated cells, indium‐dependent changes of *CDH1* and *SNAI1* mRNA expression were not affected by BAY 11‐7082, an NF‐*κ*B inhibitor, suggesting that NF‐*κ*B activation may be dispensable for indium‐induced EMT. Fibroblast‐like morphological characteristics, such as actin stress fiber formation and cell elongation, along with deconstruction of cell–cell adhesion complexes, were observed in treated cells. Overall, our study is the first to demonstrate that EMT is caused by indium compounds. This will contribute biologically to understanding the mechanism of EMT induction and clinically to unveiling the pathophysiology of indium lung disease.

## Abstract 2

Epidemiological and experimental studies have demonstrated indium‐induced pulmonary disorders, such as interstitial pneumonia and lung cancer, in humans and laboratory animals; however, the toxic mechanisms of indium compounds remain unclear. Here, we revealed that A549 cells, a human alveolar cell line, treated with indium chloride exhibited significant changes in the expression of epithelial–mesenchymal transition (EMT) markers, including E‐cadherin and SNAIL. Moreover, fibroblast‐like morphological characteristics were found in treated cells. Collectively, this study provides experimental evidence for indium‐induced EMT.

## 1 Introduction

Indium–tin oxide (ITO), composed of 90% indium oxide (In_2_O_3_) and 10% tin oxide, is an indium compound that is popular as a sintered material and in high demand for extensive applications in liquid crystal displays of televisions, computers, and mobile phones (U.S. Geological Survey 2023). Industrial workers involved in cutting, grinding, and polishing processes are at risk of exposure to indium compounds, such as ITO, In_2_O_3_, and indium hydroxide, by inhalation (Omae et al. 2011). Following the first case of indium‐related interstitial pneumonia in a Japanese facility (Homma et al. 2003), several pulmonary disorder cases in indium‐processing workers have been reported in the United States, China, Korea, Taiwan, and Japan (Choi et al. 2015; Cummings et al. 2010; Homma et al. 2005; Tsao et al. 2021; Xiao et al. 2010). Histopathological examination revealed fibrosis, proteinosis, and cholesterol granuloma in the lung tissues of individuals exposed to indium compounds (Chonan et al. 2019). Currently, this condition is referred to as “indium lung.” In experimental studies, rodents were exposed to related compounds, such as ITO, In_2_O_3_, indium phosphide (InP), and indium chloride (InCl_3_), by inhalation, resulting in not only pulmonary inflammation and fibrosis but also lung cancer (Blazka et al. 1994; Hiraku et al. 2025; Nagano et al. 2011; National Toxicology Program 2001; Tanaka et al. 2010). Consistent with these reports, a few cases of lung cancer have been reported among indium workers (Nakano et al. 2019), suggesting the potential carcinogenicity of indium compounds in humans. In vitro studies using human epithelial cells have revealed indium toxicities, such as DNA damage and activation of inflammatory signaling (Ahmed et al. 2020; Badding et al. 2015; Tabei et al. 2016); however, information about the molecular mechanism underlying the pathophysiology of indium‐induced lung diseases is extremely limited.

Epithelial tissues consist of cells that are tightly linked to each other through cell–cell adhesion complexes, which contribute to the formation of a barrier to environmental perturbations such as chemicals and pathogens (Garcia et al. 2018). As cell adhesion plays a pivotal role in the structural integrity and specific functions of organs, abnormalities in epithelial adhesion are strongly associated with the molecular etiology of multiple diseases, including cancer (Bruner and Derksen 2018). Epithelial–mesenchymal transition (EMT) is a cellular process involving the loss of epithelial characteristics and acquisition of mesenchymal characteristics. EMT was first identified in chick embryogenesis in the late 1970s (Yang et al. 2020). Since then, through evidence accumulated to date, endogenous factors (e.g., growth factors and cytokines) have been shown to activate intracellular signaling, which, in turn, induces the upregulation of core EMT‐transcription factors (TFs) along with the downregulation of adhesion molecules, leading to the deconstruction of cell–cell adhesion complexes. Specifically, multiple regulatory pathways, including NF‐*κ*B, SMAD, and STAT signaling, increase the expression of the *SNAI1* gene, which encodes core‐EMT‐TF SNAIL. This step is followed by a decrease in E‐cadherin encoded by *CDH1*, which is a hallmark of EMT. Subsequently, the cytoarchitecture changes dramatically toward a fibroblast‐like morphology through reorganization of the cytoskeleton, such as the destruction of cortical actin filaments and the formation of actin stress fibers (Lamouille et al. 2014). Notably, although EMT is crucial for wound healing, tissue regeneration, and embryogenesis, it also contributes to the pathophysiology of fibrosis and cancer (Nieto et al. 2016).

Following industrialization, a plethora of studies focusing on workers and animals exposed to heavy metals has uncovered various symptoms and mechanisms of occupational diseases. Intriguingly, heavy metals function as exogenous factors that induce EMT in the lungs. This process is considered intertwined with the molecular mechanisms of the resultant pulmonary toxicity (Chen et al. 2022). For instance, decreased expression of E‐cadherin, together with an increase in Vimentin—a mesenchymal marker—was found in the lung tissues of patients with chronic obstructive pulmonary disease (COPD) exhibiting an inverse relationship between blood cadmium concentration and respiratory function (Zheng et al. 2021). Prolonged treatment with cadmium chloride (CdCl_2_) resulted in EMT profiles involving decreased E‐cadherin, increased Vimentin, and actin stress fiber formation through the activation of regulatory pathways in A549 human alveolar epithelial cells (Fujiki et al. 2017). In another study, mice exposed to nickel chloride (NiCl_2_) developed pulmonary fibrosis, where E‐cadherin and Vimentin were down‐ and upregulated, respectively, along with activation of Smad signaling (Cao et al. 2024). Similar to the in vivo results, A549 cells treated with NiCl_2_ displayed changes in E‐cadherin and Vimentin expression through SMAD signaling activation (Yu et al. 2023). Notably, nickel compounds are implicated in the development of pulmonary diseases, such as COPD, fibrosis, and cancer, suggesting that nickel‐induced EMT partly contributes to their onset (Lee et al. 2021). Overall, a large number of epidemiological, clinical, and biological studies support the idea that EMT plays an important role in the pathophysiology of pulmonary diseases caused by heavy metals. However, EMT profiles have not been carefully assessed in the case of exposure to indium compounds; therefore, we examined whether EMT is induced in A549 cells by treatment with InCl_3_, a compound which has long been used to evaluate indium toxicity (Ahmed et al. 2020; Blazka et al. 1994; Suzuki and Yoshikawa 1976; Tabei et al. 2016).

## 2 Materials and Methods

### 2.1 Chemicals, Reagents, and Cells

**FIGURE 1:**
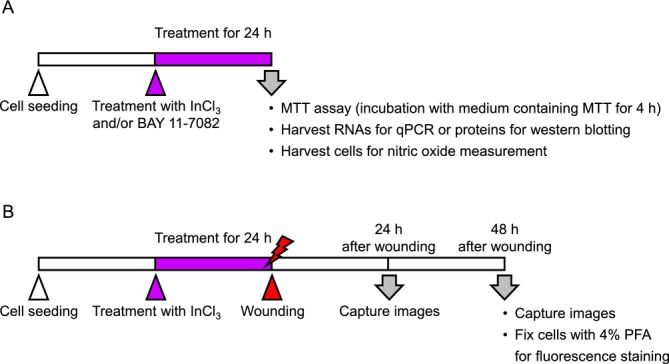
Timeline of chemical treatment and analyses. (A) Treatment with InCl_3_ and/or BAY 11‐7082 for 24 h, followed by MTT assay, qPCR, western blotting, and nitric oxide measurement in A549 cells corresponding to Figures 2, 3, 4 and S1. (B) Wound healing assay after InCl_3_ treatment for 24 h in A549 cells corresponding to Figures 5, 6, S2, and S3. Images of cells at 0, 24, and 48 h after wounding were captured to measure gap areas and compare gap closure between the untreated and treated cells. Finally, the cells were fixed with 4% paraformaldehyde (PFA) for fluorescence staining.

The chemicals and reagents used in this study are listed in Table S1. A549 cells (RIKEN BioResource Center, Tsukuba, Japan) were cultured in Dulbecco's Modified Eagle Medium (DMEM) containing 5% (v/v) fetal bovine serum (FBS) and 100‐mg/L kanamycin in 5% CO_2_ at 37°C. InCl_3_ was dissolved in autoclaved water. The experimental timeline is presented in Figure 1A,B.

### 2.2 MTT Assay

A549 cells (1 × 10^4^ cells/well) were cultured in a 96‐well plate overnight and treated with InCl_3_ at doses of 0–2000 μg/mL for 24 h. After treatment, the culture supernatant was quickly removed, and the cells were incubated with 0.5 mg/mL Thiazolyl blue tetrazolium bromide (MTT) in culture medium for 4 h at 37°C, followed by treatment with dimethyl sulfoxide (DMSO) for 10 min at room temperature. The absorbance of each well was measured at 570 nm wavelength using a Multiskan FC microplate reader (Thermo Scientific, Tokyo, Japan).

### 2.3 Quantitative PCR (qPCR)

A549 cells (2 × 10^6^ cells/dish) were cultured in 6‐cm dishes overnight and treated with InCl_3_ at doses of 0, 125, 250, and 500 μg/mL and/or 5 μM BAY 11‐7082 for 24 h. The BAY 11‐7082 concentration was reported to be effective in inhibiting NF‐*κ*B signaling in several cultured cell lines, including A549 cells (Gergen et al. 2020). Total RNA was rapidly isolated from each dish using an RNeasy Mini Kit after 24‐h treatment. cDNA for a given mRNA was synthesized using oligo‐dT and random hexamers with a PrimeScript RT reagent kit. Gene expression was determined quantitatively using a StepOnePlus Real‐Time PCR system (Life Technologies, Osaka, Japan) with Thunderbird SYBR qPCR Mix. The primers used for the genes are listed in Table S2. The mRNA expression levels were calculated using the ΔΔC_t_ method and normalized to *GAPDH* mRNA expression.

### 2.4 Western Blotting

A549 cells (2 × 10^6^ cells/dish) were cultured in 6‐cm dishes overnight and treated with 0 and 500 μg/mL InCl_3_ for 24 h. After treatment, cells were rapidly lysed with RIPA buffer containing 1 mM phenylmethylsulfonyl fluoride and were subsequently centrifuged at 16,400 *g* and 4°C for 10 min. After harvesting the supernatant, the protein concentration was measured using Coomassie Protein Assay Reagent, and 10 μg of total protein in 4X SDS Sample Buffer was loaded onto SuperSep Ace 5%–20% SDS‐PAGE gels at 500 V and 40 mA for 45 min. Proteins were transferred to PVDF membranes at 12 V and 800 mA for 30 min. The membranes were washed with Tris‐buffered saline (TBS) containing 0.1% Tween 20 (TBST) and blocked with 5% skim milk in TBST at room temperature for 1 h. Primary antibodies for E‐cadherin, ZO1, SNAIL, Vimentin, and β‐actin (1:3000 in TBST) were incubated at 4°C overnight. After incubation with secondary antibodies (1:10000 in TBST) at room temperature for 30 min, signals were visualized using ECL Prime Western Blotting Detection Reagents and detected using an ImageQuant LAS 4000 (Fujifilm, Tokyo, Japan). Band intensity in each protein on captured images was quantified using the Fiji/ImageJ software (National Institute of Health, Bethesda, MD, USA) and normalized to β‐actin.

### 2.5 Nitric Oxide Measurement

A549 cells (6 × 10^5^ cells/well) were cultured in 6‐well plates overnight and treated with 0 and 500 μg/mL InCl_3_ and/or 5 μM BAY 11‐7082 for 24 h. Cells were rapidly collected from each well after 24‐h treatment and centrifuged to remove the supernatant. The cells were treated with 4‐amino‐5‐methylamino‐2′,7′‐difuluorofluorescein diacetate (DAF‐FM) solution to assess the level of nitric oxide (NO). The stock solution was diluted in an FBS‐free medium to a final concentration of 2.5 μM. After incubation at 37°C for 15 min, the supernatant was removed by centrifugation, and the cells were resuspended in phosphate buffered saline (PBS; pH 7.4). Finally, the fluorescence intensity was measured using a FACSCantoII flow cytometer (BD Biosciences, San Jose, CA, USA).

### 2.6 Wound Healing Assay

A549 cells (4 × 10^4^ cells/chamber) were cultured in eight‐chamber culture slides (BD Falcon, Franklin Lakes, NJ, USA) overnight and treated with InCl_3_ for 24 h. After washing with PBS, the cells were wounded with a P200 tip and incubated with InCl_3_‐free medium. Photos of the cells were taken at 0, 24, and 48 h after wounding using an All‐in‐One Microscope BZ‐X800 (Keyence, Osaka, Japan). Fluorescence staining was performed at 48 h, as described below. Gap areas in each image were measured using the Fiji/ImageJ software to calculate gap closure (%; the ratio of the gap areas at 24 or 48 to 0 h).

### 2.7 Fluorescence Staining

After 48‐h incubation, the cells were promptly washed with PBS and then fixed with 4% paraformaldehyde (PFA)/PBS (w/v) at room temperature for 10 min, followed by permeabilization with 0.1% Triton X‐100/PBS (v/v). The cells were treated with 5% bovine serum albumin (BSA)/PBS (w/v) for 1 h and incubated with primary antibodies for E‐cadherin and ZO1 (1:100 in 5% BSA/PBS) at 4°C overnight. After washing with PBS, the cells were incubated with secondary antibodies (1:500 in 5% BSA/PBS) and Phalloidin‐iFluor 647 (1:1000) at room temperature for 1 h. Finally, each slide was covered with SlowFade Diamond Antifade Mountant containing DAPI. Fluorescence‐stained images were captured using the All‐in‐One Microscope BZ‐X800. Subcellular localization of E‐cadherin and ZO1 was quantitatively evaluated through bin analysis. Briefly, areas between the two nuclei of neighboring cells were equally divided into 10 bins, and the fluorescent intensity in each bin was measured using the Fiji/ImageJ software (80 cells from four chambers/group) to compare the distribution of relative values (the lowest value among 10 bins = 1) between treated and untreated cells.

### 2.8 Statistical Analysis

Statistical analysis was performed using BellCurve for the Excel software (Social Survey Research Information, Tokyo, Japan). The MTT assay, qPCR, western blotting, NO measurement, wound healing assay, and E‐cadherin and ZO1 localization were analyzed using Student's *t* test or one‐way analysis of variance (ANOVA), followed by the Tukey–Kramer multiple comparison test, and *p* values of < 0.05 were considered statistically significant. Normality and variance homogeneity tests were not conducted in the present study.

## 3 Results

### 3.1 Toxic Responses in A549 Cells Treated With InCl_3_

**FIGURE 2:**
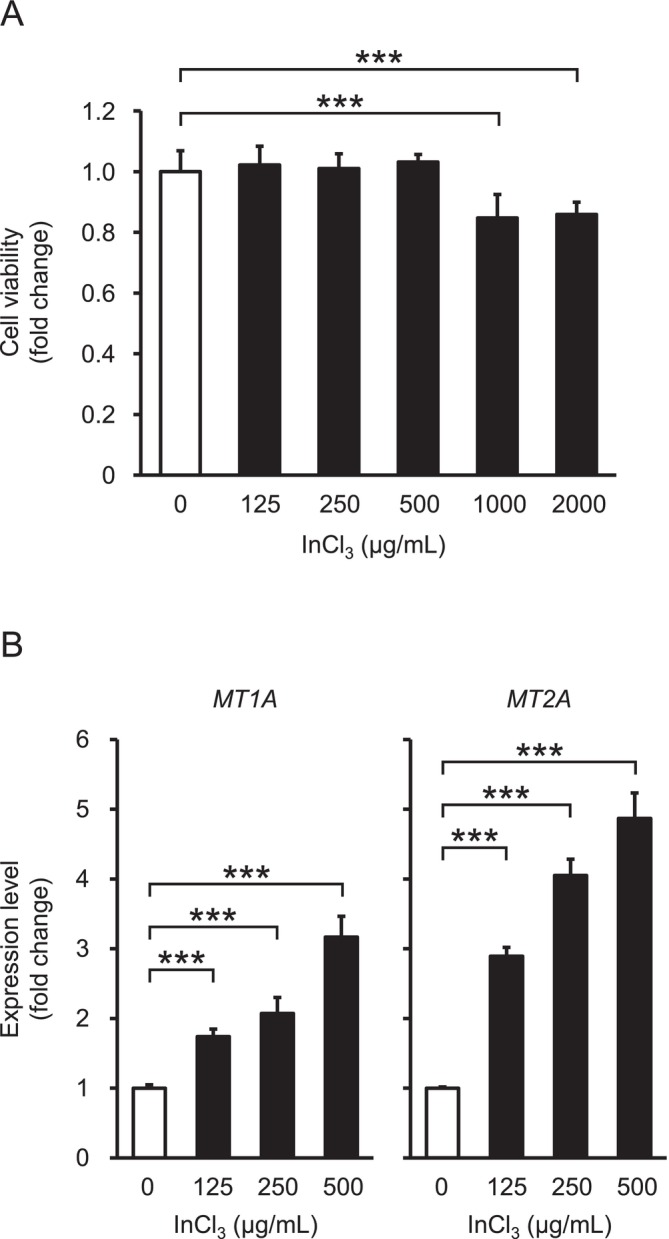
Toxic responses in A549 cells treated with InCl_3_. (A) Cell viability in treatments at doses of 0, 125, 250, 500, 1000, and 2000 μg/mL for 24 h, with ratio in 0 μg/mL set as 1.0 (*n* = 8 wells/group). (B) Expression levels of *MT1A* and *MT2A* mRNAs in treatments at doses of 0, 125, 250, and 500 μg/mL, with ratio in 0 μg/mL set as 1.0 (*n* = 4 dishes/group). Values represent the mean ± SD. *** indicates *p* values of < 0.001 (vs. 0 μg/mL; Tukey–Kramer multiple comparison test).

Initially, an MTT assay was performed to examine the viability of A549 cells treated with InCl_3_ at doses of 0–2000 μg/mL for 24 h. Results revealed a significant decrease in their viability at 1000 and 2000 μg/mL (Figure 2A); thus, 500 μg/mL or less was chosen for subsequent experiments. Because the induction of metallothioneins (MTs) occurs typically in response to heavy metals, such as cadmium and lead (Waalkes et al. 1984), the expression levels of *MT* genes in treated cells were analyzed by qPCR. In line with previous in vivo and in vitro studies (Suzuki and Yoshikawa 1976; Tabei et al. 2016), the significant upregulation of *MT1A* and *MT2A* mRNAs was identified at 125, 250, and 500 μg/mL dose‐dependently (Figure 2B), suggesting that indium ion influx activates intracellular signaling for metal metabolism.

### 3.2 Changes of EMT Marker Expression in A549 Cells Treated With InCl_3_

**FIGURE 3:**
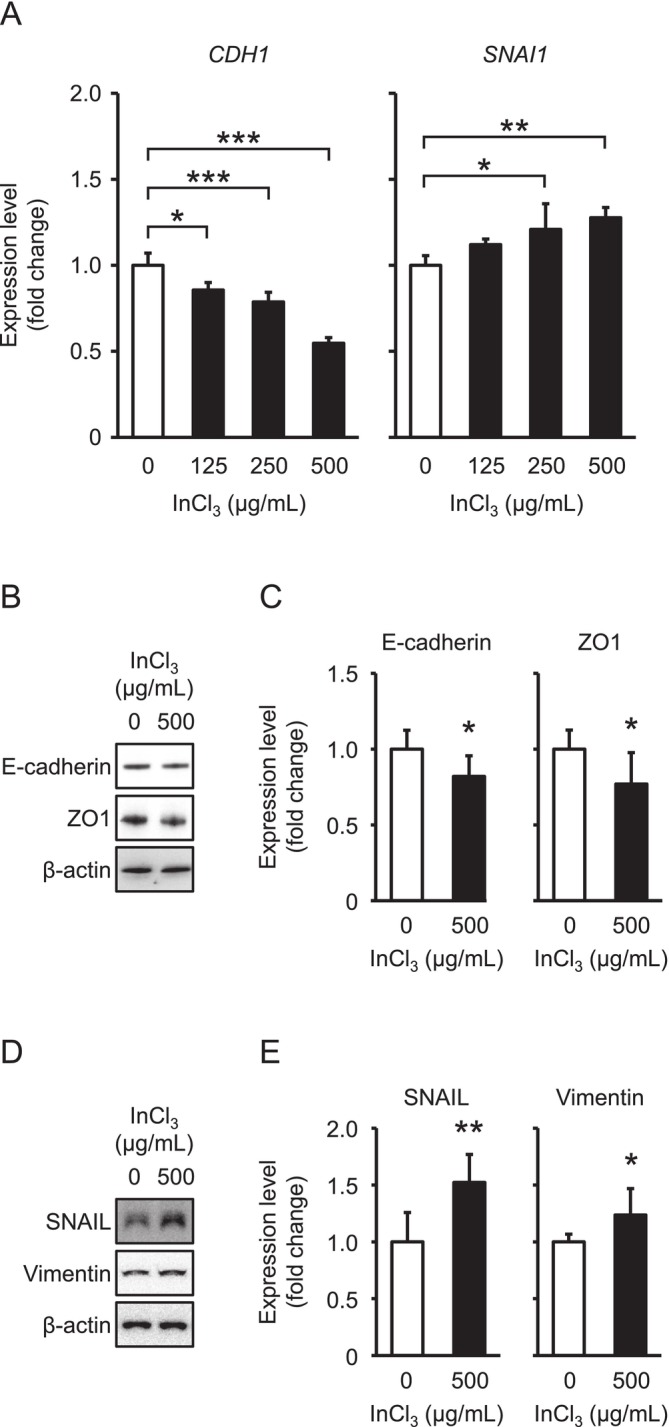
Changes of EMT marker expression in A549 cells treated with InCl_3_. (A) Expression levels of *CDH1* and *SNAI1* mRNAs in treatments at doses of 0, 125, 250, and 500 μg/mL, with ratio in 0 μg/mL set as 1.0 (*n* = 4 dishes/group). (B–E) Expression levels of epithelial (E‐cadherin and ZO1; B, C) and mesenchymal (SNAIL and Vimentin; D, E) marker proteins in treatments at 0 and 500 μg/mL, with ratio in 0 μg/mL set as 1.0 (*n* = 6 dishes/group). Representative images of each protein (B, D) and quantification of their expression levels normalized by *β*‐actin (C, E). Values represent the mean ± SD. *, **, and *** indicate *p* values of < 0.05, < 0.01, and < 0.001, respectively (vs. 0 μg/mL; Tukey–Kramer multiple comparison test or Student's *t* test).

Next, the expression levels of EMT marker genes were quantitatively analyzed by qPCR, and comparisons were performed between untreated and treated cells. Significant downregulation of *CDH1* mRNA was found at doses of 125, 250, and 500 μg/mL; conversely, *SNAI1* mRNA was significantly upregulated at 250 and 500 μg/mL (Figure 3A). Considering these findings, western blotting was subjected to comparison of epithelial and mesenchymal proteins between 0 and 500 μg/mL, resulting in a decrease in E‐cadherin and ZO1, and an increase in SNAIL and Vimentin by treatment (Figures 3B–E and S1). The changes of these markers suggest that indium ion influx induces intracellular signaling associated with EMT, in addition to metal metabolism.

### 3.3 NF‐*κ*B Signaling Activation in A549 Cells Treated With InCl_3_

**FIGURE 4:**
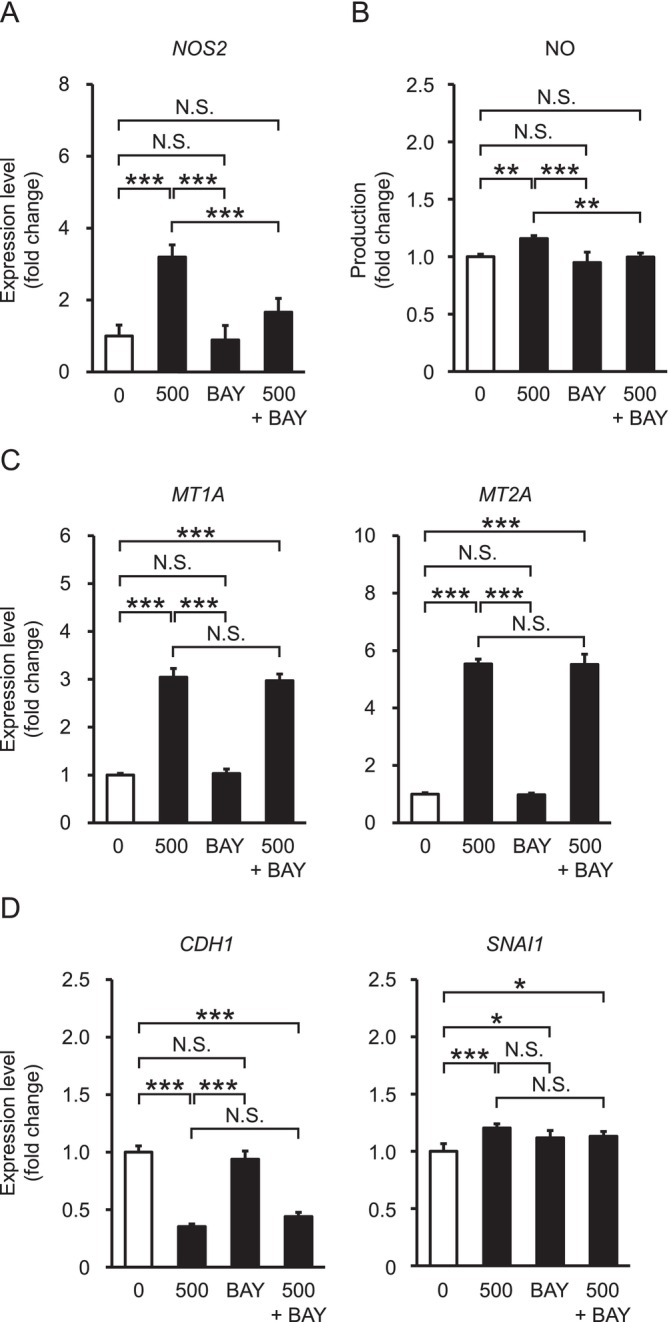
NF‐*κ*B signaling activation in A549 cells treated with InCl_3_. (A, B) *NOS2* mRNA expression (A) and NO production (B) in treatments at 0 and 500 μg/mL InCl_3_ and/or 5 μM BAY 11‐7082, with ratio in 0 μg/mL set as 1.0 (*n* = 4 dishes/group). (C, D) Expression levels of MT (*MT1A* and *MT2A*, C) and EMT marker (*CDH1* and *SNAI1*, D) mRNAs in A549 cells treated with 0 and 500 μg/mL InCl_3_ and/or 5 μM BAY 11‐7082, with ratio in 0 μg/mL set as 1.0 (*n* = 4 dishes/group). Values represent the mean ± SD. *, **, and *** indicate *p* values of < 0.05, < 0.01, and < 0.001, respectively (Tukey–Kramer multiple comparison test).NF‐*κ*B signaling activation in A549 cells treated with InCl_3_. (A, B) *NOS2* mRNA expression (A) and NO production (B) in treatments at 0 and 500 μg/mL InCl_3_ and/or 5 μM BAY 11‐7082, with ratio in 0 μg/mL set as 1.0 (*n* = 4 dishes/group). (C, D) Expression levels of MT (*MT1A* and *MT2A*, C) and EMT marker (*CDH1* and *SNAI1*, D) mRNAs in A549 cells treated with 0 and 500 μg/mL InCl_3_ and/or 5 μM BAY 11‐7082, with ratio in 0 μg/mL set as 1.0 (*n* = 4 dishes/group). Values represent the mean ± SD. *, **, and *** indicate *p* values of < 0.05, < 0.01, and < 0.001, respectively (Tukey–Kramer multiple comparison test).

To date, information about intracellular signaling crucial for the indium toxicity is limited. Nevertheless, NF‐*κ*B signaling for induction of *NOS2* expression is activated by several indium compounds, leading to nitrative DNA damage caused by NO production (Afroz et al. 2018; Ahmed et al. 2020). In accordance with previous work, significant upregulation of *NOS2* mRNA at 500 μg/mL was found also in this study, whereas co‐treatment with BAY 11‐7082, an NF‐*κ*B inhibitor, impeded indium‐dependent *NOS2* expression (Figure 4A). Consistent with the mRNA expression, NO production was significantly elevated by treatment with only InCl_3_ and not coupled with BAY 11‐7082 (Figure 4B). Conversely, neither indium‐dependent expression of *MT1A* nor that of *MT2A*, not belonging to NF‐*κ*B target genes, was affected by BAY 11‐7082 (Figure 4C). The expression levels of *CDH1* and *SNAI1* mRNAs were significantly altered by both treatment with InCl_3_ combined with BAY 11‐7082 and treatment with InCl_3_ alone, although *SNAI1* was slightly increased by only BAY 11‐7082 (Figure 4D). These results imply that, even though NF‐*κ*B was activated by indium, its signaling may be unnecessary for indium‐induced EMT.

### 3.4 Microscopic Observations of A549 Cells Treated With InCl_3_

**FIGURE 5:**
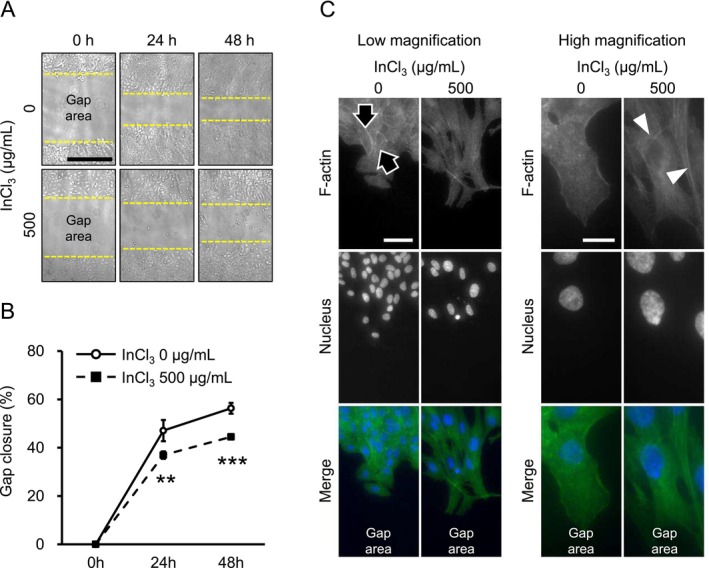
Motility and morphology of A549 cells treated with InCl_3_. (A, B) Cell motility in treatments at 0 and 500 μg/mL was evaluated by wound healing assay (*n* = 4 chambers/group). Representative images of gap areas at 0, 24, and 48 h after wounding (A) and quantification of gap closure (B). Yellow dashed lines indicate the borders between cell layers and gap areas in A. Gap closure indicates the percentage of gap area at 24 or 48 to 0 h in B. (C) Representative images of the edges of cell layers at 48 h after wounding. F‐actin (low and high magnification on left and right sides, respectively) and nuclei (DAPI) are shown as green and blue on merged images, respectively. Arrowheads and black arrows indicate actin stress fibers and cortical actin filaments, respectively, in C. Scales are as follows: 500 μm in A, and 50 and 20 μm (low and high magnification, respectively) in C. Values represent the mean ± SD. ** and *** indicate *p* values of < 0.01 and < 0.001, respectively (vs. 0 μg/mL; Student's *t* test).

**FIGURE 6:**
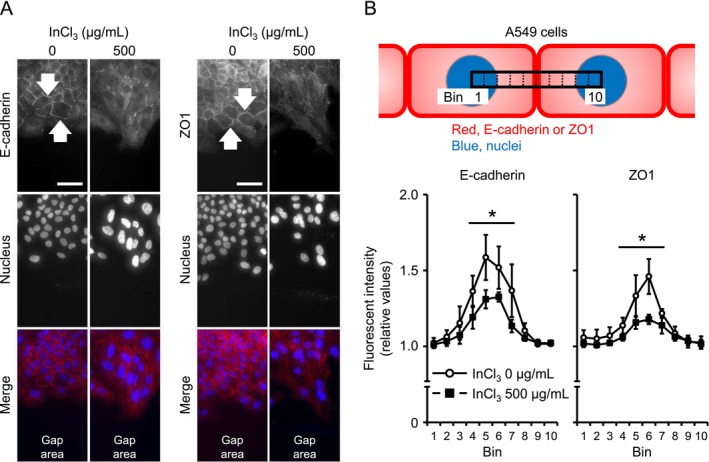
Subcellular localization of epithelial maker proteins of A549 cells treated with InCl_3_. (A) Representative images of the edges of cell layers at 48 h after wounding. Epithelial markers (E‐cadherin and ZO1) and nuclei (DAPI) are shown as red and blue on merged images, respectively. (B) Fluorescent intensity of epithelial markers in treatments at 0 and 500 μg/mL was evaluated through bin analysis, with ratio in the lowest value among 10 bins set as 1.0 (*n* = 4 chambers/group), indicating disrupted localization of E‐cadherin and ZO1 by treatment in middle bins. White arrows indicate epithelial markers. Scales are as follows: 50 μm in A. Values represent the mean ± SD. * indicates *p* values of < 0.05 (vs. 0 μg/mL; Student's *t* test).

In general, EMT contributes to increased cell migration and invasion through dynamic reorganization of cytoarchitecture and morphology (Yang et al. 2020); thus, a wound healing assay was performed to evaluate cell motility at doses of 0 and 500 μg/mL. Contrary to our hypothesis, gap closure in treated cells was significantly lower than that in untreated cells at both 24 and 48 h after wounding (Figure 5A,B). This suggests that indium decreased cell motility; however, the viability did not significantly change under incubation with InCl_3_‐free medium for 48 h following 24‐h treatment at doses of 125–1000 μg/mL (Figure S3A). Conversely, in line with our hypothesis, indium‐treated cells exhibited obvious fibroblast‐like morphological characteristics. Specifically, treatment induced destruction of cortical actin filaments as well as elongated cell morphology with actin stress fibers at the edges of cell layers (Figure 5C). In contrast, only a few untreated cells exhibited such morphological changes. Additionally, decreased gap closure and actin filament reorganization were also observed at 125 and 250 μg/mL (Figure S2A–C). Moreover, E‐cadherin and ZO1 were localized on the cell membranes of untreated cells; however, the localization of the two proteins was disrupted by treatment (Figure 6A,B), indicating deconstruction of cell–cell adhesion complexes. Interestingly, there was no significant difference in viability even at 1000 μg/mL (Figure S3A). Presumably, viability recovered through cell proliferation during incubation after InCl_3_ removal. Similar to those at lower concentrations, cell motility, cytoarchitecture, and E‐cadherin localization at 1000 μg/mL were also affected (Figure S3B–F). Overall, these results fit well with the expression patterns of EMT markers and imply that transient exposure may have been continuously eliciting EMT even after changing into the environment without indium.

## 4 Discussion

Lung biopsies of patients with idiopathic pulmonary fibrosis show increased expression of mesenchymal proteins in the alveolar epithelium, which is clinical evidence implying a relationship between EMT and fibrosis in the lungs (Marmai et al. 2011). Pulmonary fibrosis has been observed in patients with indium lung disease (Chonan et al. 2019; Cummings et al. 2012; Tsao et al. 2021) and in rodents exposed to indium compounds (Blazka et al. 1994; Hiraku et al. 2025; Nagano et al. 2011; National Toxicology Program 2001; Tanaka et al. 2010). In this study, we demonstrated that indium treatment induced not only changes in EMT marker expression at the mRNA and protein levels, but also abnormalities in the subcellular localization of E‐cadherin and ZO1. These results suggest that indium is presumably a new exogenous factor triggering EMT and that such EMT markers could be useful molecules for examining whether EMT contributes to fibrosis in indium lung disease.

Indium compounds functionally activate NF‐*κ*B to upregulate the *NOS2* gene, resulting in nitrative DNA damage through NO production (Afroz et al. 2018; Ahmed et al. 2020), which implies a pivotal role of NF‐*κ*B signaling in indium toxicity. Intriguingly, IGF‐I‐dependent activation of NF‐*κ*B signaling elicits EMT through upregulation of *SNAI1*, stabilization of SNAIL, and downregulation of E‐cadherin in human mammary epithelial cells (Kim et al. 2007). In accordance with previous findings, due to up‐ and downregulation of *SNAI1*/SNAIL and *CDH1*/E‐cadherin, respectively, under our experimental conditions, we surmised that treatment with InCl_3_ activates NF‐*κ*B signaling, ultimately leading to EMT. However, contrary to previous findings, NF‐*κ*B signaling seems dispensable for indium‐induced EMT, because inhibition of NF‐*κ*B by BAY 11‐7082 did not affect the indium‐dependent changes in EMT marker expression. As previously mentioned, the regulatory pathways underlying EMT comprise complex molecular networks (Lamouille et al. 2014). Given that inflammation is evoked by indium exposure, STAT signaling may be involved in EMT, because interleukin‐6 activates STAT, leading to EMT (Sullivan et al. 2009). In addition, NiCl_2_ reportedly affects the expression levels of EMT markers through SMAD2/3 phosphorylation in mouse lungs and A549 cells (Cao et al. 2024; Yu et al. 2023), suggesting that InCl_3_ may also activate SMAD signaling. As information about perturbed signaling in indium‐treated cells is still limited, future studies should include global analyses of gene and protein expression to identify previously unreported signaling that is crucial for indium toxicity. Based on these results, additional experiments using pharmacological and molecular biological methods (e.g., inhibitor treatment and gene knockdown/out) are important to demonstrate intracellular signaling linking indium compounds with EMT.

Notably, some heavy metals function both as EMT inducers and as carcinogens. Specifically, cadmium and nickel compounds, which are well‐known EMT inducers, belong to Group 1 (carcinogenic to humans) of the International Agency for Research on Cancer (IARC) (IARC 1990, 1993). Among the indium compounds, InP and ITO are currently classified as Group 2A (probably carcinogenic to humans) and Group 2B (possibly carcinogenic to humans), respectively (IARC 2006, 2018). The carcinogenicity of indium compounds in humans has been the subject of active debate because emerging evidence suggests that exposure to indium may be associated with the onset of lung cancer in workers (Nakano et al. 2019). Interestingly, in addition to connecting cells, E‐cadherin plays an important role in the repression of tumor development and cancer progression (Bruner and Derksen 2018). *CDH1* gene mutations encoding aberrant E‐cadherin proteins have been found in patients with cancer (Berx et al. 1995), which is experimentally supported by the phenotypes of a mouse model with enhanced tumorigenesis harboring a dominant negative form of E‐cadherin (Dahl et al. 1996). In the present study, we found the downregulation of *CDH1*/E‐cadherin expression and deconstruction of cell–cell adhesion complexes composed of E‐cadherin in indium‐treated cells. Consistent with the notion of indium carcinogenicity, our results suggest the possibility that E‐cadherin may be a key molecule in lung cancer caused by indium compounds.

Our results from molecular experiments suggest that EMT occurred in indium‐treated cells; however, gap closure was significantly reduced, which is not in accordance with the general features of EMT. Although this result implies a reduction in cell motility, there is another possibility that cell proliferation is disrupted by treatment. Because, in addition to motility, proliferation reportedly contributes to gap closure in wound healing assays (Sakaue‐Sawano et al. 2008), the reduced gap closure of treated cells in the present study may be caused by disrupted proliferation on the edges of cell layers. In support of this speculation, not only reduced gap closure but also cell cycle arrest was observed in A549 cells treated with linalool and 1,8‐cineole, which are plant‐derived isoprenoids (Rodenak‐Kladniew et al. 2020). Thus, further studies are needed to examine whether exposure to indium compounds affects cell proliferation and cell cycle.

It is worth noting that the range of concentrations of utilized indium compounds was rather specific. The concentrations for examining EMT profiles were determined based on the cell viability inferred from the MTT assay (i.e., 125–500 μg/mL) and were of a similar level as those in previous studies (i.e., 200–400 μg/mL) (Gwinn et al. 2013; Tabei et al. 2016). However, low‐concentration effects have attracted considerable attention because DNA damage by indium compounds—even at concentrations as low as 5 ng/mL to 50 μg/mL—has been reported in recent studies (Afroz et al. 2018; Ahmed et al. 2020). In our previous study, we estimated that 5 ng/mL of indium compounds in vitro would have a similar level in the lungs of ITO workers based on airborne indium concentration, alveolar deposition, and occupational environment (Ahmed et al. 2020). In contrast, the present study aimed to examine whether indium compounds can induce EMT even at high concentrations because, to the best of our knowledge, there is no information on EMT induction in an indium‐dependent manner. Our experimental conditions, which involved relatively high concentrations, demonstrated that EMT was at least partly induced by indium treatment, while cell motility was decreased by transient treatment. Certain in vitro studies on EMT induction by heavy metals have used longer treatment periods (i.e., several days or weeks) (Chen et al. 2022). Thus, future studies should examine whether EMT is elicited at lower concentrations (i.e., 5 ng/mL or less) and longer treatments with indium compounds.

Indium compounds have long been recognized as safe materials; however, accumulating evidence suggests pulmonary toxicity in ITO workers and laboratory animals. In line with this notion, we provide experimental evidence of indium‐induced EMT. Consistent with the guideline of the Japan Ministry of Health, Labour and Welfare (Ministry of Health, Labour and Welfare 2010), our findings support the importance of reducing indium exposure in workplaces and detecting indium lung disease as early as possible.

In summary, because research on indium‐induced health issues is less advanced than that for other toxic metals, little is known about the pathophysiology of indium lung disease and the molecular mechanism of indium toxicity. This study successfully revealed that indium compounds elicit EMT in alveolar epithelial cells, suggesting that disrupted expression of molecules linked with EMT could potentially be a part of the toxic mechanism of indium lung disease. Further studies focusing on indium‐induced EMT will offer valuable insights into the pathophysiology and etiology of pulmonary disorders, leading to a deeper understanding of the mechanisms underlying the effects of toxic metals.

## Author Contributions

Conceptualization: E.K. and Y.H.; investigation: E.K., S.A., and H.C.; methodology: E.K., S.A., and H.C.; funding acquisition: E.K.; supervision: E.K.; writing – original draft: E.K. and Y.H.

## Conflicts of Interest

The authors declare no conflicts of interest.

## Supporting information


**Table S1.** Chemicals and reagents.
**Table S2.** Primer sequences used for qPCR.
**Figure S1. Original images of western blotting**. Epithelial markers (E‐cadherin and ZO1, A), mesenchymal markers (SNAIL and Vimentin, B), and β‐actin were detected in A549 cells treated with InCl_3_ at doses of 0 and 500 μg/mL. The red dashed boxes in A and B correspond to Figure 3B and 3D, respectively.
**Figure S2. Motility and morphology of A549 cells at doses of 125, 250, and 500 μg/mL**. (A, B) Cell motility in treatments was evaluated by wound healing assay (*n* = 4 chambers/group). Representative images of gap areas at 0 and 48 h after wounding (A) and quantification of gap closure (B). Yellow dashed lines indicate the borders between cell layers and gap areas in A. Gap closure indicates the percentage of gap area at 48 h to 0 h in B. (C) Representative images of the edges of cell layers at 48 h after wounding. F‐actin and nuclei (DAPI) are shown as green and blue on merged images, respectively. Arrowheads and black arrows indicate actin stress fibers and cortical actin filaments, respectively, in C. Scales are as follows: 500 μm in A and 50 μm in C. Values represent the mean ± SD *, **, and *** indicate *p*‐values of < 0.05, <0.01, and < 0.001, respectively (vs. 0 μg/mL; Tukey–Kramer multiple comparison test).
**Figure S3. EMT characteristics of A549 cells treated with InCl**_**3**_ **at a dose of 1000 μg/mL**. (A) Cell viability in treatments at doses of 0, 125, 250, 500, and 1000 μg/mL for 24 h, followed by incubation with InCl_3_‐free medium for 48 h, with ratio in 0 μg/mL set as 1.0 (*n* = 8 wells/group; one‐way ANOVA, *p* > 0.05). (B, C) Cell motility in treatments at 0 and 1000 μg/mL was evaluated using wound healing assay (*n* = 4 chambers/group). Representative images of gap areas at 0 and 48 h after wounding (B) and quantification of gap closure (C). Yellow dashed lines indicate the borders between cell layers and gap areas in B. Gap closure indicates the percentage of gap area at 48 h to 0 h in C. (D, E) Representative images of the edges of cell layers at 48 h after wounding. F‐actin (D), E‐cadherin (E), and nuclei (DAPI) are shown as green, red, and blue on merged images, respectively. (F) Fluorescent intensity of E‐cadherin was analyzed through bin analysis, with ratio in the lowest value among 10 bins set as 1.0 (*n* = 4 chambers/group), indicating disrupted localization of E‐cadherin by treatment in middle bins. The arrowhead, black arrow, and white arrow in D and E indicate actin stress fibers, cortical actin filaments, and E‐cadherin, respectively. Scales are as follows: 500 μm in B, and 50 μm in D and E. Values represent the mean ± SD. * and *** indicate *p*‐values of < 0.05 and < 0.001, respectively (vs. 0 μg/mL; Student’s *t*‐test).

## Data Availability

Data available on request from the corresponding author.
