# Supplementary material for: Epithelial–Mesenchymal Transition in Human Alveolar Cells Exposed to Indium Chloride
Source: J Appl Toxicol. 2025 Jul 7;45(11):2353–62. doi: 10.1002/jat.4848 (PMC12488300; doi:10.1002/jat.4848)
Supplement: Supplementary file 1 — Table S1. Chemicals and reagents. Table S2. Primer sequences used for qPCR. Figure S1. Original images of western blotting. Epithelial markers (E‐cadherin and ZO1, A), mesenchymal markers (SNAIL and Vimentin, B), and β‐actin were detected in A549 cells treated with InCl3 at doses of 0 and 500 μg/mL. The red dashed boxes in A and B correspond to Figure 3B and 3D, respectively. Figure S2. Motility and morphology of A549 cells at doses of 125, 250, and 500 μg/mL. (A, B) Cell motility in treatments was evaluated by wound healing assay (n = 4 chambers/group). Representative images of gap areas at 0 and 48 h after wounding (A) and quantification of gap closure (B). Yellow dashed lines indicate the borders between cell layers and gap areas in A. Gap closure indicates the percentage of gap area at 48 h to 0 h in B. (C) Representative images of the edges of cell layers at 48 h after wounding. F‐actin and nuclei (DAPI) are shown as green and blue on merged images, respectively. Arrowheads and black arrows indicate actin stress fibers and cortical actin filaments, respectively, in C. Scales are as follows: 500 μm in A and 50 μm in C. Values represent the mean ± SD *, **, and *** indicate p‐values of < 0.05, <0.01, and < 0.001, respectively (vs. 0 μg/mL; Tukey–Kramer multiple comparison test). Figure S3. EMT characteristics of A549 cells treated with InCl 3 at a dose of 1000 μg/mL. (A) Cell viability in treatments at doses of 0, 125, 250, 500, and 1000 μg/mL for 24 h, followed by incubation with InCl3‐free medium for 48 h, with ratio in 0 μg/mL set as 1.0 (n = 8 wells/group; one‐way ANOVA, p > 0.05). (B, C) Cell motility in treatments at 0 and 1000 μg/mL was evaluated using wound healing assay (n = 4 chambers/group). Representative images of gap areas at 0 and 48 h after wounding (B) and quantification of gap closure (C). Yellow dashed lines indicate the borders between cell layers and gap areas in B. Gap closure indicates the percentage of gap area at 48 h to 0 h in C. (D, E) [file JAT-45-2353-s001.pdf]

Supporting information

Epithelial–mesenchymal transition in human alveolar cells exposed to indium chloride

Eiki Kimura, Sharif Ahmed, Haijiao Chen, Yusuke Hiraku

Department of Environmental Health, University of Fukui School of Medical Sciences

Correspondence

Eiki Kimura, Department of Environmental Health, University of Fukui School of Medical Sciences, 23-3 Matsuoka-shimoaizuki, Eiheiji, Fukui 910-1193, Japan.

Tel: +81-776-61-8337

E-mail: kimuraei@u-fukui.ac.jp

Table S1. Chemicals and reagents.

Product name	Company (address)	Cat. No.
4-Amino-5-methylamino-2',7'-difluorofluorescein diacetate (DAF-FM)	Goryo Chemical (Sapporo, Japan)	SK1003-01
Anti-β-actin antibody	Santa Cruz Biotechnology (Santa Cruz, CA, USA)	sc-47778
Anti-E-cadherin antibody	Cell Signaling Technology (Danvers, MA, USA)	3195
Anti-Mouse IgG HRP conjugated	Cell Signaling Technology (Danvers, MA, USA)	7076
Anti-Rabbit IgG Alexa Fluor 568 conjugated	Invitrogen (Eugene, OR, USA)	A11036
Anti-Rabbit IgG HRP conjugated	Cell Signaling Technology (Danvers, MA, USA)	7074
Anti-SNAIL antibody	Cell Signaling Technology (Danvers, MA, USA)	3879
Anti-Vimentin antibody	Cell Signaling Technology (Danvers, MA, USA)	5741
Anti-ZO1 antibody	Cell Signaling Technology (Danvers, MA, USA)	8193
BAY 11-7082	Sigma-aldrich (St. Louis, MO, USA)	B5556
Bovine serum albumin (BSA)	Sigma-aldrich (St. Louis, MO, USA)	A3059
Coomassie Protein Assay Reagent	Thermo Scientific (Rockford, IL, USA)	1856209
Difco Skim milk	Becton, Dickinson and Company (Sparks, MD, USA)	232100
Dimethyl sulfoxide (DMSO)	FUJIFILM Wako Pure Chemical Corporation (Osaka, Japan)	045-24511
Dulbecco's Modified Eagle Medium (DMEM) (Low Glucose)	Nacalai Tesque (Kyoto, Japan)	08456-36
ECL Prime Western Blotting Detection Reagents	Cytiva (Tokyo, Japan)	RPN2232
Fetal bovine serum (FBS)	Biowest (Nuaillé, France)	S1810
Immobilon-P transfer membranes (PVDF membrane)	Merck Millipore (Tullagreen, Carrightwohill, Co. Cork, Ireland)	IPVH00010
Indium (III) chloride Tetrahydrate	Kanto chemical (Tokyo, Japan)	20286-33
Kanamycin	FUJIFILM Wako Pure Chemical Corporation (Osaka, Japan)	113-00701
NuPAGE LDS Sample buffer (4X)	Thermo Fisher Scientific (Waltham, MA, USA)	NP0007
PageRuler Prestained Protein Ladder	Thermo Scientific (Rockford, IL, USA)	26616
Paraformaldehyde (PFA)	Wako pure chemical (Osaka, Japan)	160-00515
Phalloidin-iFluor 647	AAT Bioquest (Pleasanton, CA, USA)	23127
Phenylmethylsulfonyl Fluoride (PMSF)	Nacalai Tesque (Kyoto, Japan)	27327-81

Polyethylene glycol mono-p-isooctylphenyl ether (Triton X-100)	Nacalai Tesque (Kyoto, Japan)	12969-25
PrimeScript RT reagent Kit	Takara (Kusatsu, Japan)	RR037A
RIPA buffer	Cell Signaling Technology (Danvers, MA, USA)	9806
RNeasy Mini Kit	Qiagen (Tokyo, Japan)	74104
SlowFade Dioamond Antifade Mountant with DAPI	Invitrogen (Eugene, OR, USA)	S36964
SuperSep Ace 5–20%	FUJIFILM Wako Pure Chemical Corporation (Osaka, Japan)	292-36411
Thiazolyl blue tetrazolium bromide (MTT)	Sigma-aldrich (St. Louis, MO, USA)	M2128
Thunderbird SYBR qPCR Mix	TOYOBO (Osaka, Japan)	QPS-201

Table S2. Primer sequences used for qPCR.

Gene symbol	Forward	Reverse
CDH1	5′-ataatcctccgatcttcaatc-3′	5′-tttcagtgtggtgattacg-3′
GAPDH	5′-catgagaagtatgacaacag-3′	5′-gataccaaagttgtcatgga-3′
MT1A	5′-ctcgaagatatagaaagagtga-3′	5′-agcaacttttattatcattcaca-3′
MT2A	5′-tcccagatgtaaagaacgc-3′	5′-aaaggaatatagcaaacggtc-3′
NOS2	5′-tcaagctatcgaatttgtca-3′	5′-aggttcctgttgtttctatc-3′
SNAI1	5′-taatccagagtttaccttcc-3′	5′-cagatgagcattggcag-3′

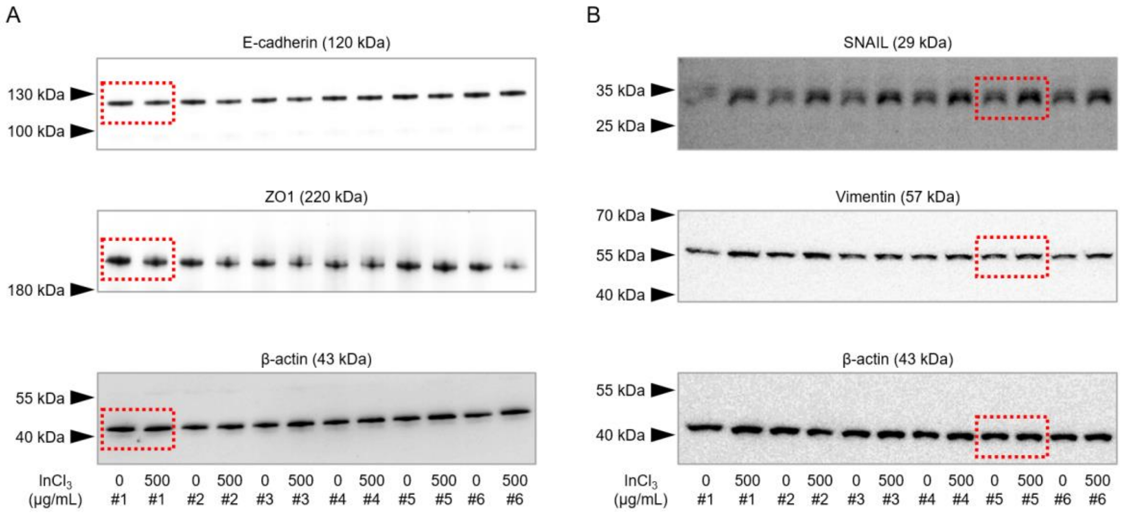

Figure S1. Original images of western blotting. Epithelial markers (E-cadherin and ZO1, A), mesenchymal markers (SNAIL and Vimentin, B), and β-actin were detected in A549 cells treated with $InCl_3$ at doses of 0 and 500 μg/mL. The red dashed boxes in A and B correspond to Fig. 3B and 3D, respectively.

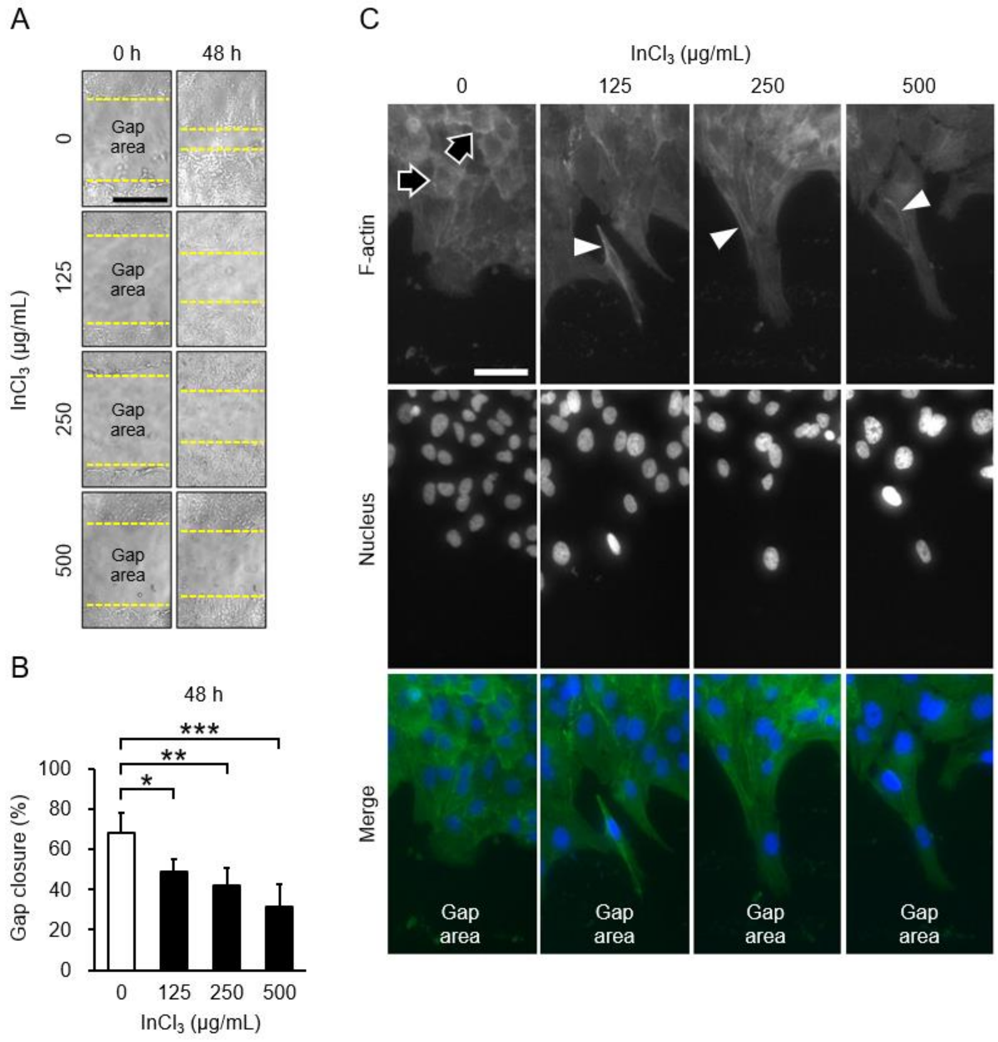

Figure S2. Motility and morphology of A549 cells at doses of 125, 250, and 500 µg/mL. (A, B) Cell motility in treatments was evaluated by wound healing assay (n = 4 chambers/group). Representative images of gap areas at 0 and 48 h after wounding (A) and quantification of gap closure (B). Yellow dashed lines indicate the borders between cell layers and gap areas in A. Gap closure indicates the percentage of gap area at 48 h to 0 h in B. (C) Representative images of the edges of cell layers at 48 h after wounding. F-actin and nuclei (DAPI) are shown as green and blue on merged images, respectively. Arrowheads and black arrows indicate actin stress fibers and cortical actin filaments, respectively, in C. Scales are as follows: 500 µm in A and 50 µm in C. Values represent the mean ± SD. *, **, and *** indicate p-values of <0.05, <0.01, and <0.001, respectively (vs. 0 µg/mL; Tukey–Kramer multiple comparison test).

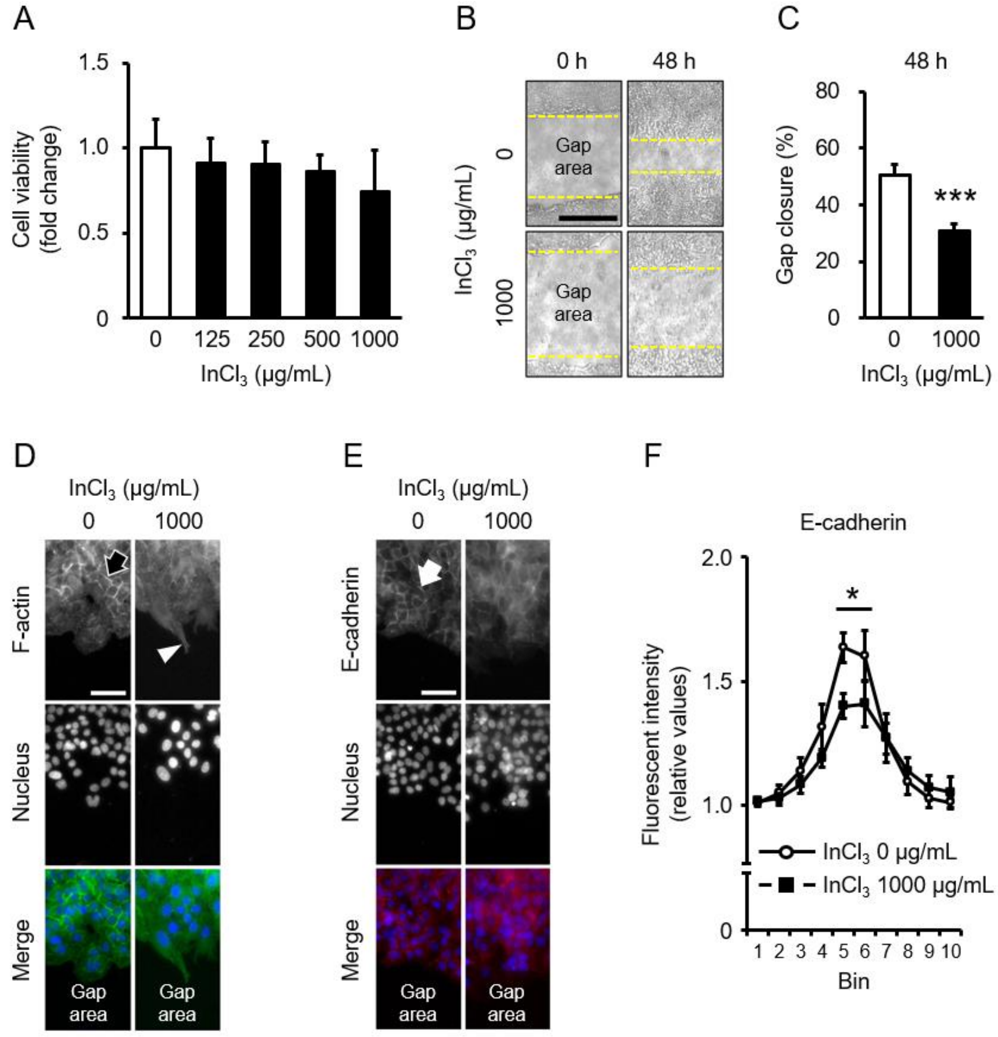

Figure S3. EMT characteristics of A549 cells treated with $InCl_3$ at a dose of 1,000 µg/mL. (A) Cell viability in treatments at doses of 0, 125, 250, 500, and 1,000 µg/mL for 24 h, followed by incubation with $InCl_3$-free medium for 48 h, with ratio in 0 µg/mL set as 1.0 (n = 8 wells/group; one-way ANOVA, $p > 0.05$). (B, C) Cell motility in treatments at 0 and 1,000 µg/mL was evaluated using wound healing assay (n = 4 chambers/group). Representative images of gap areas at 0 and 48 h after wounding (B) and quantification of gap closure (C). Yellow dashed lines indicate the borders between cell layers and gap areas in B. Gap closure indicates the percentage of gap area at 48 h to 0 h in C. (D, E) Representative images of the edges of cell layers at 48 h after wounding. F-actin (D), E-cadherin (E), and nuclei (DAPI) are shown as green, red, and blue on merged images, respectively. (F) Fluorescent intensity of E-cadherin was analyzed through bin analysis, with ratio in the lowest value among 10 bins set as 1.0 (n = 4 chambers/group), indicating disrupted localization of E-cadherin by treatment in middle bins. The arrowhead, black arrow, and white arrow in D and E indicate actin stress fibers, cortical actin filaments, and E-cadherin, respectively. Scales are as follows: 500 µm in B, and 50 µm in D and E. Values represent the mean ± SD. * and *** indicate p-values of <0.05 and <0.001, respectively (vs. 0 µg/mL; Student's t-test).
